# Low CD36 and LOX-1 Levels and* CD36* Gene Subexpression Are Associated with Metabolic Dysregulation in Older Individuals with Abdominal Obesity

**DOI:** 10.1155/2016/5678946

**Published:** 2016-07-25

**Authors:** Perla-Monserrat Madrigal-Ruíz, Rosa-Elena Navarro-Hernández, Sandra-Luz Ruíz-Quezada, Fernanda-Isadora Corona-Meraz, Mónica Vázquez-Del Mercado, Eduardo Gómez-Bañuelos, Jorge Castro-Albarran, Flavio Sandoval-García, Luis-Javier Flores-Alvarado, Beatriz-Teresita Martín-Marquez

**Affiliations:** ^1^Departamento de Biología Molecular y Genómica, Centro Universitario de Ciencias de la Salud, Universidad de Guadalajara, Sierra Mojada No. 950, Colonia Independencia, 44340 Guadalajara, JAL, Mexico; ^2^Instituto de Investigación en Reumatología y del Sistema Músculo Esquelético, Centro Universitario de Ciencias de la Salud, Universidad de Guadalajara, Sierra Mojada No. 950, Colonia Independencia, 44340 Guadalajara, JAL, Mexico; ^3^UDG-CA-701, Grupo de Investigación Inmunometabolismo en Enfermedades Emergentes (GIIEE), Centro Universitario de Ciencias de la Salud, Universidad de Guadalajara, Sierra Mojada No. 950, Colonia Independencia, 44340 Guadalajara, JAL, Mexico; ^4^Departamento de Farmacobiología, Centro Universitario de Ciencias Exactas e Ingenierías, Universidad de Guadalajara, Boulevard Marcelino García Barragán No. 1421, 44430 Guadalajara, JAL, Mexico; ^5^Servicio de Reumatología, División de Medicina Interna, Hospital Civil “Dr. Juan I. Menchaca”, Universidad de Guadalajara, Salvador de Quevedo y Zubieta No. 750, 44340 Guadalajara, JAL, Mexico; ^6^UDG-CA-703, Grupo de Investigación en Inmunología y Reumatología, Centro Universitario de Ciencias de la Salud, Universidad de Guadalajara, Sierra Mojada No. 950, Colonia Independencia, 44340 Guadalajara, JAL, Mexico; ^7^Departamento de Clínicas Médicas, Antiguo Hospital Civil de Guadalajara, Calle Hospital No. 320, Colonia El Retiro, 44360 Guadalajara, JAL, Mexico

## Abstract

*Background*. Obesity study in the context of scavenger receptors has been linked to atherosclerosis. CD36 and LOX-1 are important, since they have been associated with atherogenic and metabolic disease but not fat redistribution. The aim of our study was to determinate the association between CD36 and LOX-1 in presence of age and abdominal obesity.* Methods*. This is a cross-sectional study that included 151 healthy individuals, clinically and anthropometrically classified into two groups by age (<30 and ≥30 years old) and abdominal obesity (according to World Health Organization guidelines). We excluded individuals with any chronic and metabolic illness, use of medication, or smoking. Fasting blood samples were taken to perform determination of CD36 mRNA expression by real-time PCR, lipid profile and metabolic and low grade inflammation markers by routine methods, and soluble scavenger receptors (CD36 and LOX-1) by ELISA.* Results*. Individuals ≥30 years old with abdominal obesity presented high atherogenic index, lower soluble scavenger receptor levels, and subexpression of* CD36* mRNA (54% less). On the other hand, individuals <30 years old with abdominal adiposity presented higher levels in the same parameters, except LOX-1 soluble levels.* Conclusion*. In this study, individuals over 30 years of age presented low soluble scavenger receptors levels pattern and* CD36* gene subexpression, which suggest the chronic metabolic dysregulation in abdominal obesity.

## 1. Introduction

Obesity is characterized by progressive enlargement of adipose tissue [1, 2]. It has been shown that the fat distribution rather than total amount of fat is associated with increased cardiovascular disease (CVD) risk [3]. Since CD36 is a cell surface glycoprotein that belongs to class B scavenger receptor family, it is considered as multifunctional receptor: in adipocyte it acts as a fatty acid translocase of long-chain fatty acids; however, in monocyte/macrophage, it regulates oxLDL uptake [4]. On the other hand, LOX-1 is classified among E-type scavenger receptor family, is expressed in vascular endothelium, and recognizes oxLDL, cellular debris, and several structurally unrelated molecules as a ligand; it is highly expressed in the cardiovascular system by proinflammatory and prooxidative stimuli [5, 6].

LOX-1 and CD36 favor the development of atherosclerosis through their ability to bind and internalize modified LDL facilitating formation of macrophage foam cells and their location to subendothelial space promoting endothelial dysfunction and CVD.

Additionally, several studies associate an increase of soluble form of CD36 (sCD36) in symptomatic carotid stenosis with metabolic disorders, such as type 2 diabetes mellitus (T2DM). Meanwhile, the soluble form of LOX-1 (sLOX-1) has been mainly associated with diverse atherogenic diseases like arterial stiffness, T2DM, and myocardial infarction and it is even proposed as a biomarker for acute coronary syndrome [7–18]. Several studies showed an inflammatory paracrine interaction between adipocytes and macrophages which could be dependent on scavenger receptors; this pathogenic process involves a state of dysmetabolic profile and chronic inflammation which contributes to abdominal obesity development [19, 20].

However, the association of sCD36 and sLOX-1 levels with fat redistribution and metabolic markers is not known. The aim of our study was to determine the association between CD36 and LOX-1 in presence of age and abdominal obesity.

## 2. Materials and Methods

### 2.1. Subjects' Selection and Classification

In this cross-sectional study, we recruited 151 adults aged between 20 and 59 years of general population of Mexico. They were classified according to the recommendations of World Health Organization Expert Consultation, by WC, waist-hip ratio (WHR), and waist-to-height ratio (WHtR), into two groups: the first group includes individuals with abdominal obesity, if any of the following conditions were present: WC ≥ 90.0 cm, WHR ≥ 0.90, and WHtR ≥ 0.525 in men and WC ≥ 80.0 cm, WHR ≥ 0.80, and WHtR ≥ 0.530 in women; the second group includes individuals without abdominal obesity, a group which has lower values of these measurements. In relation to age, an age of 30 years was established as a cutoff and two groups were stablished: <30 years old and ≥30 years old.

We consider additional criteria for disease risk associations: BMI from 18.50 to 24.99 kg/m^2^ was considered without disease risk; for increased disease risk: BMI 25.00 to 29.99 kg/m^2^ plus WC <102.0 cm (in men) or <88.0 cm (in women) was considered; for high disease risk, we consider WC ≥102.0 cm (in men) or ≥88.0 cm (in women) and BMI ≥30.0 kg/m^2^ plus any WC measurement was classified as very high disease risk [21–23].

We included individuals who at the time of the study did not present with glucose intolerance, infectious diseases, hypertension, pregnancy, anemia, diagnosis of CVD, malignancy, and renal and metabolic diseases such as T2DM. We excluded subjects with current medication, smoking, or drugs use.

For ethics conduct before enrolment, participants were informed about the study and signed a consent form following Helsinki Declaration guidelines [24], and the institutional (Guadalajara University) review boards committees ensured appropriate ethical and biosecurity conduct.

### 2.2. Subjects' Medical History and Physical Examination

All individuals who satisfied inclusion criteria were clinically evaluated by a physician who performed a complete medical history; assessment of general health status and vital signs were included: blood pressure (executed 3 times with the subject in the sitting position and relaxing for 15 minutes before the measurement), heart, respiratory rate, and body temperature.

### 2.3. Subjects' Body Fat Storage Measurements

We evaluated the following body measurements: height was measured to the nearest 1 mm by using a stadiometer (seca GmbH & Co. KG., Hamburg, Germany) and body weight, total body fat mass, and trunk body fat mass (absolute, kg, and relative, %) were determined by using bioelectrical impedance analysis (TANITA BC-418 segmental body composition analyzer, Tokyo, Japan) to the nearest 0.1 kg. WC, hip circumference (HC), and coronal abdominal diameter were measured by using an anthropometric fiberglass tape (GULICK® length 0–180 cm precision ±0.1, USA). At the level of the iliac crest (L4-5), sagittal abdominal diameter was measured using a sliding-beam, abdominal caliper (precision ±0.1 cm, Holtain Ltd., Crosswell, Crymych, Pembs., SA41 3UF, UK) with the patient lying in a supine position in the examination table [25–29]. Five measures of skinfold thicknesses (i.e., biceps, triceps, subscapular, suprailiac, and abdominal) were obtained on the right side of the body by using a Harpenden skinfold caliper (opened 80 mm and precision of ±0.2 mm, constant pressure: 10 g/mm^2^; Holtain Ltd., Crosswell, Crymych, Pembs., SA41 3UF, UK). All these measurements were carried out by the same anthropometrist in duplicate following the procedures recommended by anthropometric indicators measurement guide [30, 31].

To determine obesity and adiposity indexes, the following math calculations were used, BMI, kg/m^2^ = weight (kg)/height^2^ (m); WHR = WC cm/HC cm; WHtR = WC cm/height (cm); conicity index (CI) = WC (cm)/0.109√weight (kg)/height (cm); total adipose area (TAA, cm^2^) = WC^2^/4*π*; visceral area (VA, cm^2^) = *π*(WC/2*π* − abdominal skinfold)^2^; subcutaneous abdominal area (cm^2^) = TAA − VA [26]; visceral adipose index (VAI): for males, VAI = (WC/36.58 + (1.896*∗*BMI))6(TG/0.81)6(1.52/HDLc), and for females, VAI = (WC/39.68 + (1.886*∗*BMI))6(TG/1.03)6(1.31/HDLc); abdominal volume index (AVI, L) = [2 × WC^2^ + (0.7 cm)(WC − HC)^2^/1000] [32]; and homeostasis model assessment-insulin resistance (HOMA-IR) = [basal glucose mg/dL × (basal insulin *μ*UI/mL)/405] [33, 34], in addition to the sum of the 5 skin fold thicknesses (ST5) [35].

### 2.4. Metabolic and Inflammatory Markers and Scavenger Receptors Levels Measurements

We confirmed overnight fast of 12 hours in all subjects and obtained two venous blood samples, with and without anticoagulant (PAXgene® Blood RNA Tube CAT. 762165 and BD SST*™* Plus −13 mm CAT. 368159, BD Diagnostic Systems Montenegro 1402 (C1427AND), Buenos Aires, Argentina, resp.). Samples were allowed to clot for 30 minutes at 20°C before centrifugation for 10 minutes/20°C at 1509 RCF (Rotanta 460R, Andreas Hettich GmbH & Co. KG.); serum was collected and stored immediately at −86°C until further analysis.

We quantified serum concentration of basal glucose, mg/dL, and lipid profile, mg/dL, which included triglycerides, total cholesterol, HDLc, LDLc, and VLDLc [36] (high, low, and very low density lipoprotein cholesterols, resp.), apolipoproteins A1 and B (Apo-A1 and Apo-B), mg/dL, free fatty acids (FFA, mmol/mL), serum C3, mg/dL, high sensitivity CRP with a limit of detection of 0.15 mg/L, with routine colorimetric, enzymatic, and immunoturbidimetry methods (Randox Laboratories, 55 Diamond Road, Crumlin Co. Antrim, Northern Ireland, UK), and erythrocyte sedimentation rate (ESR, mm/h) by Wintrobe method [37].

Through using commercial enzyme-linked immunosorbent assays (ELISA), we determined soluble levels of insulin sensitivity of 0.399 *μ*UI/mL (ALPCO, 26-G Keewaydin Drive, Salem, NH 03079), sCD36 sensitivity of 312.5 ng/mL, and sLOX-1 sensitivity of 5.0 pg/mL (AVISCERA BIOSCENCE, INC., 2348 Walsh Avenue, Suite C, Santa Clara, CA 95051). Based on the lipid profile measurements, we calculated the following atherogenic indexes: TC/HDLc, LDLc/HDLc, TG/HDLc, and Apo-A1/Apo-B.

### 2.5. CD36 mRNA Expression Analysis

Mononuclear cells from the subjects were isolated by density gradient media with separating solution Lymphoprep*™* (AXIS-SHIELD, P.O. Box 6863, Rodelokka, 0504 Oslo, Norway) [38]. Purified mononuclear cells were used directly for total RNA isolation; it was performed according to the manufacturer's procedure using TRIzol® LS Reagent (Ambion, Invitrogen*™*, by Life Technologies, 5791 Van Allen Way, Carlsbad, CA 92008) based on the single-step RNA isolation modified method reported by Chomczynski and Sacchi [39, 40]. The complementary DNA synthesis (cDNA) was performed with 2 *μ*g of each total RNA sample using a reaction size of 20 *μ*L with oligo (dT) 18-primer (100 ng/*μ*L), RNase-free, DEPC treated water and Moloney Murine Leukemia Virus Reverse Transcriptase (M-MLV RT) kit (Applied Biosystems, 850 Lincoln Centre Drive, Foster City, CA 94404) and stored at −20°C until being used for expression analyses.

Real-time quantitative polymerase chain reaction (qPCR) was conducted using the StepOne detection system, EXPRESS SYBR® GreenER*™* qPCR SuperMix Universal, and sequence detector software (Applied Biosystems, 850 Lincoln Centre Drive, Foster City, CA 94404) was used for data analysis. A threshold cycle (C_T_) value was determined from each amplification plot.

In brief,* CD36* mRNA expression was performed in a final reaction volume of 20 mL (40 *μ*M forward and reverse primer, 50 nM ROX, 2x SYBR Green qPCR master mix and cDNA 100 ng). The conditions of the reaction were as follows: holding at 95°C/10 min, cycling (35 cycles of 95°C/15 s and 60°C/60 s), and melt curve at 95°C/15 s, 60°C/60 s, and 95°C/15 s. Expression of target genes was normalized by the endogenous reference gene* GAPDH*; sequence specific primers were forward: 5′-GGAGCGAGATCCCTCCAAAAT-3′ [41] and reverse: 5′-GGCTGTTGTCATACTTCTCATGG-3′ and for* CD36* target gene they were forward: 5′-CTATGCTGTATTTGAATCCGACGTT-3′ [42] and reverse: 5′-CCTGTGTACATTTCACTTCCTCATT-3′.

The relative expression fold changes of target genes were calculated using the comparative C_T_ method with 2^−ΔΔC_T_^ equation [43]. For quality control, a blank and samples previously confirmed as positive for each gene were used as controls. In addition, all samples were identified in duplicate by two different analysts. The expression success rate was 100%.

### 2.6. Statistical Analysis

Data were analyzed with statistics software SPSS v21 (IBM Inc., Chicago, IL, USA) and with GraphPad Prism v6.01 (2014 Inc., 2236 Beach Avenue, Jolla, CA 92037). Results are given as mean ± standard deviation (SD). The data distribution of clinical and laboratory variables was evaluated with *Z* Kolmogorov-Smirnov test; parametric and nonparametric tests were performed as appropriate. An age and gender (male or female) adjusted ANCOVA analysis was made in individuals included in the study. The clinical and laboratory characteristics of the study group were analyzed with unpaired Student's *t*-test or Mann-Whitney *U* test to compare quantitative data in two groups. Data from serum concentrations of scavenger receptors, laboratorial assessment, and adiposity variables were subjected to Pearson's correlation tests. A two-tailed *P* value less than 0.05 was considered statistically significant.

## 3. Results

### 3.1. Study Subjects Show Increased Body Fat Storage and Cardiovascular Disease Risk

We included 151 individuals that were classified by age and presence of abdominal obesity; 70% were females. The anthropometric measures are shown in Table 1. Seventy percent presented overweight (mean BMI: 29.88 ± 3.3 kg/m^2^), 37% of these individuals were categorized with CVD risk, almost 70% of the group were older than ≥30 years (data no shown), and 78% were identified with an increased body fat storage.

When comparing subgroups with abdominal obesity, we observed a similar distribution of trunk fat storage in young and older individuals, while older subjects presented higher measurements on triceps, subscapular, and suprailiac skinfold thickness compared to younger subjects (Table 1).

Additionally, in the abdominal obesity subgroup, we observed increased WHtR in older (0.619 ± 0.054) versus younger (0.574 ± 0.042) individuals (*P* = 0.019).

### 3.2. Individuals with Abdominal Obesity Presented Subclinical Inflammatory State and Alterations of Lipids Profile

**Figure 1:**
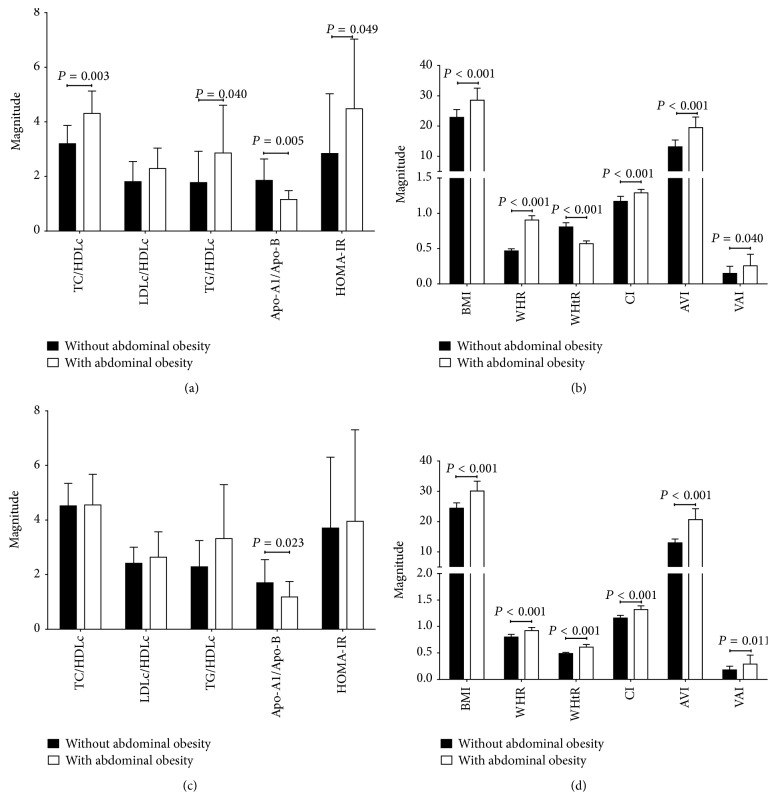
Metabolic and adiposity indexes in study group. Subjects without abdominal obesity: *n* = 33; subjects with abdominal obesity: *n* = 118. Data are presented as mean ± SD. (a) and (b) Individuals <30 years old: Mann-Whitney *U* test comparing the groups with abdominal obesity versus individuals without abdominal obesity. (c) and (d) Individuals ≥30 years old with abdominal obesity versus individuals without abdominal obesity. TC: total cholesterol; HDLc, LDLc, and VLDLc: high density lipoprotein cholesterol, low density lipoprotein cholesterol, and very low density lipoprotein cholesterol, respectively; Apo: apolipoprotein; HOMA-IR: homeostasis model assessment-insulin resistance; BMI: body mass index; BFR: body fat ratio; WHtR: waist-to-height ratio; CI: conicity index; AVI: abdominal volume index; VAI: visceral adipose index.

We found that individuals below 30 years of age with abdominal obesity showed alterations on basal glucose, total cholesterol, HDLc levels, and proinflammatory profile; this same scenario was seen in older individuals with similar adiposity characteristics in which a decreased level of Apo-A1 and HDLc was also found (Table 2). Those individuals with abdominal obesity, regardless of age, presented higher measurements of abdominal adiposity and atherogenic indexes (Figure 1).

### 3.3. Age and the Presence of Abdominal Obesity Have a Detrimental Effect on sCD36 and sLOX-1 Levels and Increase of Metabolic Markers

The subgroup of individuals below 30 years of age compared with the subgroup of individuals over 30 years of age showed an increase in accumulation of abdominal fat deposits in association with a dyslipidemic profile, which was in accordance with an increase in atherogenic indexes and metabolic markers (Figures 1(a) and 1(c) and Table 2).

**Figure 2:**
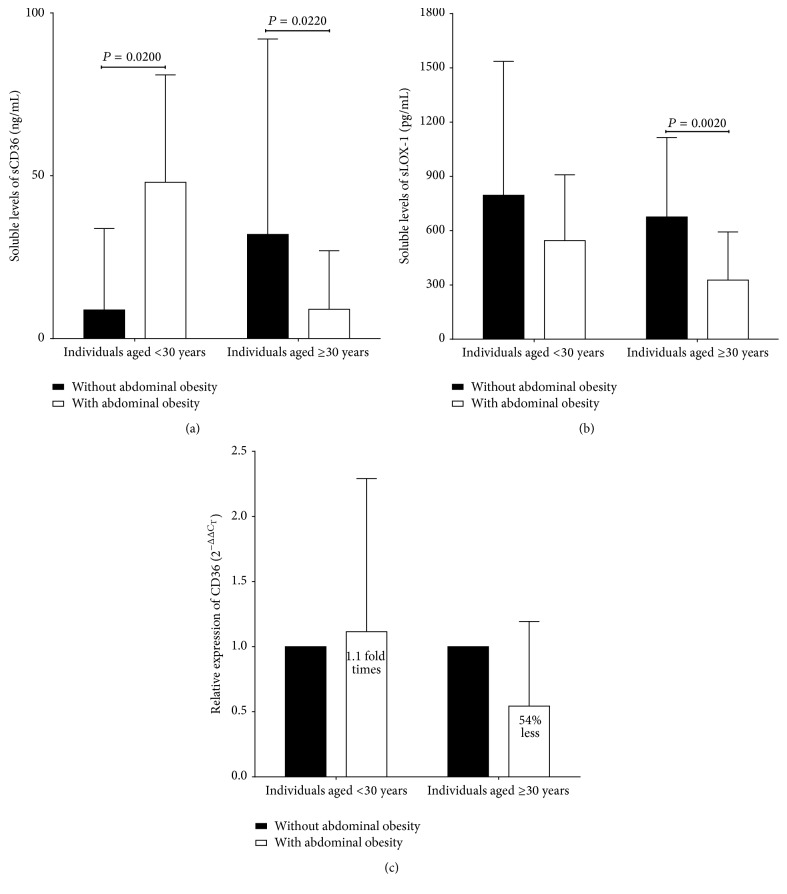
Scavenger receptors LOX-1 and sCD36 expression levels in study group. Subjects without abdominal obesity: *n* = 86; subjects with abdominal obesity: *n* = 54. Data are presented as mean ± SD in individuals below 30 years of age and individuals above 30 years of age. (a) Soluble levels of CD36. (b) Soluble levels of LOX-1. (c) mRNA expression levels of CD36. LOX-1: lectin like oxidized low density lipoprotein receptor 1.

We observed that individuals over 30 years of age with abdominal obesity presented lower sCD36 levels compared to individuals without abdominal obesity, while individuals aged below 30 years showed higher levels (Figure 2(a)). On the other hand, levels of sLOX-1 were lower in both groups (Figure 2(b)). A more detailed analysis was performed, in which 21% (*β* = 67,802.625;  *P* = 0.001) of sCD36 levels are affected by sex, and 35% (*β* = −3,891.21;  *P* = 0.006) are affected by the combination of sex and abdominal skinfold thickness on an age adjusted ANCOVA.

### 3.4. CD36 Relative Expression Is Associated with Abdominal Obesity

We observed in young individuals with abdominal obesity a slight increase in relative expression (1.11 fold times versus individuals without abdominal obesity); on the other hand, the group over 30 years of age with abdominal obesity showed subexpression (54% less, Figure 2(c)).

### 3.5. Adiposity Is Associated with Scavenger Receptors Levels, Low-Grade Systemic Inflammatory Markers, and Body Fat Storage Indexes

In the case of sLOX-1 and FFA levels, a positive correlation with sCD36 was observed, in contrast with the negative correlations in both scavenger receptors with abdominal adiposity and body fat mass distribution and inflammatory and metabolic markers which are displayed in Table 3.

## 4. Discussion

Abdominal obesity is generated when there is excess accumulation of adipose tissue in a progressive manner, preferably in the central region of the body. In this setting, we evaluated scavenger receptors production with metabolic, inflammatory, and adiposity profiles in two groups of adults without clinical manifestations of disease. We found a significant percent of individuals with high risk of CVD associated with the presence of abdominal obesity [44]. Although BMI is a useful tool to identify individuals at risk, there are other criteria that help recognize earlier CVD risk in an age-independent manner [22, 23].

In the context of this study, clinical and anthropometric profile in individuals below 30 years of age versus individuals over 30 years of age suggests that abdominal obesity is independent of aging, which involves different phenotypes observed in several clinical stages during development of the pathogenic process.

In our study, all individuals with abdominal obesity showed increased adiposity indexes and low-grade inflammation status with dyslipidemic profile. It has been reported that accumulation of fat mass in abdominal area presents diverse subclinical alterations characterized by changes on lipid and carbohydrate metabolism [25, 45]. All these changes are due to the development of low-grade inflammation. This process starts an abnormal mechanism with the following sequential steps: the monocytes from the bloodstream migrate to adipose tissue; these cells polarize to M1 macrophages and secrete proinflammatory cytokines that can act locally in paracrine way favoring the low-grade inflammation, which is redundantly perpetuated [20, 45].

Although age is an important factor in the development of metabolic alterations, a remarkable observation is that, in presence of abdominal obesity, our younger group presented high atherogenic indexes compared to its corresponding older group.

We also noticed that the accumulation of abdominal fat leads to higher fasting glucose and dyslipidemia observed in individuals with abdominal obesity. These evidences confirm that the abdominal obesity can be a modifiable cardiovascular risk factor, unlike other metabolic diseases such as atherosclerosis, hypertension, and T2DM [46].

Soluble CD36 levels have been reported not only in patients with carotid stenosis and unstable atheromatous plaque but also in chronic inflammatory related states such as diabetic nephropathy, polycystic ovary syndrome, and severity of hepatic steatosis and insulin resistance and individuals with morbid obesity [47–50].

In our study we observed in individuals below 30 years of age increased levels of sCD36 in parallel with body fat deposits predominately on abdominal area. These results contrast with the opposite case observed in older individuals, where sCD36 levels decreases with age and presence of abdominal obesity. A consistent elevation of sCD36 in patients with T2DM, insulin resistance, and obesity has been documented; after bariatric surgery in adults, sCD36 levels decline and metabolic markers improve [8, 10, 14]; meanwhile, there are reports of low receptor membrane expression on peripheral blood mononuclear cells in women with obesity and pharmacologically treated rheumatoid arthritis patients with subclinical atherosclerosis [51, 52]. Based on these reports, we decided to measure scavenger receptor levels in individuals with abdominal obesity.

Our results showed two subsets of* CD36* expression in soluble levels related to age below and above 30 years, while a low soluble LOX-1 pattern in older individuals with abdominal adiposity negative correlates with several adiposity indexes. On the contrary, we observed positive correlations between FFA and sLOX-1 levels with sCD36 which suggest the interaction of these scavenger receptors in states of pathological adipose tissue distribution.

Increased sLOX-1 levels have been associated with acute coronary syndrome or T2DM patients that denote their contribution in atherosclerosis development [53–55]. In our study we showed an alternative scenario to what is reported in previous studies in subjects with metabolic diseases; our findings could be explained in part by a strict compliance on the inclusion criteria used in our study and by taking into account abdominal obesity indicators. Even though there is a functional redundancy in scavenger receptors it seems that in undefined circumstances they work as coreceptors to display several responses in different clinical context; this interplay between receptors has not been completely elucidated [56].

While CD36 soluble receptor levels are considered an early marker of insulin resistance, it could also be considered that modulation of scavenger receptor could represent a precompensation mechanism in individuals with an altered metabolism. Based on our results, we could hypothesize that low soluble CD36 levels and their gene subexpression may be an early homeostatic state previous to the establishment of metabolic syndrome, thereby reinforcing the central role of dysfunctional adipose tissue as a trigger for the metabolic complications associated with long-term obesity. The multiple functions and the heterogeneity of cells that express this receptor make it difficult to isolate the exact role of these receptors in diverse clinical contexts.

## 5. Conclusions

In this study, individuals over 30 years of age presented low soluble scavenger receptors levels pattern and* CD36* gene subexpression, which suggest the chronic metabolic dysregulation in abdominal adiposity.

## Figures and Tables

**Table 1:** Body dimensions and distribution of body fat storage according to age and abdominal obesity.

	Study group			
	<30 years old		≥30 years old	
	Without abdominal obesity	With abdominal obesity	Without abdominal obesity	With abdominal obesity
*n*	21	24	12	94
Female (%)	14 (67)	11 (45)	10 (83)	71 (75)
Age (years)	23 ± 3	24 ± 3	41 ± 9	45 ± 8
BMI (kg/m^2^)	22.8 ± 2.4	28.5 ± 3.8	24.4 ± 1.7	30.0 ± 3.3
*Measurements*				
Height (cm)	169.7 ± 8.6	170.5 ± 9.0	160.6 ± 6.2	163.2 ± 9.1
Body weight (kg)^*∗*^	65.9 ± 9.6	83.3 ± 15.1	62.9 ± 6.0	80.3 ± 12.4
Total body fat mass (kg)^*∗*^	15.2 ± 6.2	26.3 ± 7.8	19.5 ± 4.2	28.7 ± 7.0
Body fat mass (%)^*∗*^	23.2 ± 8.6	31.9 ± 8.1	30.8 ± 2.2	35.6 ± 6.8
Waist circumference (cm)^*∗*^	79.7 ± 6.9	98.5 ± 8.4	79.4 ± 4.3	101.0 ± 8.7
Hip circumference (cm)^*∗*^	99.1 ± 5.6	108.4 ± 5.9	99.4 ± 4.8	109.5 ± 7.6
Trunk body fat mass (kg)^*∗*^	7.5 ± 3.5	14.5 ± 4.1	9.4 ± 2.4	14.5 ± 3.8
Trunk body fat mass (%)^*∗*^	11.3 ± 4.6	17.4 ± 3.4	14.7 ± 3.3	18.0 ± 3.5
Total adipose area (cm^2^)^*∗*^	510 ± 85	778 ± 135	503 ± 54	817 ± 145
Subcutaneous abdominal area (cm^2^)^*∗*^	381 ± 140	579 ± 185	489 ± 45	604 ± 253
Visceral area (cm^2^)	301 ± 586	267 ± 277	456 ± 769	435 ± 699
Sagittal abdominal diameter (cm)^*∗*^	17.8 ± 2.1	21.7 ± 2.6	18.6 ± 1.1	23.0 ± 3.7
Coronal abdominal diameter (cm)^*∗*^	28.2 ± 4.1	33.8 ± 4.9	28.9 ± 3.8	31.5 ± 6.7
Skinfold thickness (mm)				
Biceps	7.8 ± 4.5	9.5 ± 4.2	9.4 ± 4.4	12.9 ± 5.2
Triceps^*∗*^	16.0 ± 5.1	29.1 ± 30.3	17.8 ± 4.1	21.6 ± 6.3
Subscapular^*∗*^	15.2 ± 5.3	20.4 ± 8.6	17.1 ± 5.1	23.9 ± 12.8
Suprailiac^*∗*^	13.6 ± 5.7	19.6 ± 8.1	15.0 ± 7.1	20.8 ± 6.7
Abdominal	18.5 ± 7.9	18.9 ± 9.5	20.5 ± 9.2	23.8 ± 8.8
ST5^*∗*^	70.5 ± 25.0	97.7 ± 47.2	80.0 ± 26.0	103.3 ± 29.7

*n* = 151. Data are presented as mean ± SD. Mann-Whitney *U* test with ^*∗*^ *P* < 0.001 comparing groups by age: individuals with abdominal obesity versus individuals without abdominal obesity. BMI: body mass index; ST5: sum of five measures of skinfold thicknesses (i.e., biceps, triceps, subscapular, suprailiac, and abdominal).

**Table 2:** Metabolic and inflammation markers in individuals included in the study.

Measurements	Study group			
	<30 years old		≥30 years old	
	Without abdominal obesity	With abdominal obesity	Without abdominal obesity	With abdominal obesity
*n*	21	24	12	94
Basal glucose (mg/dL)^*∗*^	86 ± 12	97 ± 15	88 ± 9	98 ± 13
Basal insulin (*μ*UI/mL)	13.5 ± 9.8	18.7 ± 10.6	17.5 ± 12.8	16.1 ± 15.0
Triglycerides (mg/dL)	90 ± 41	122 ± 65	120 ± 55	146 ± 76
Total cholesterol (mg/dL)^*∗*^	166 ± 30	191 ± 38	235 ± 53	206 ± 40
HDLc (mg/dL)^*∗*^	49.0 ± 6.6	42.7 ± 6.3	51.8 ± 4.5	46.2 ± 7.3
LDLc (mg/dL)	89.4 ± 34.5	101.4 ± 33.2	126.1 ± 36.4	119.1 ± 37.3
VLDLc (mg/dL)	18.0 ± 8.3	24.4 ± 13.1	24.0 ± 11.0	29.2 ± 15.2
Apo-A1 (mg/dL)^*∗*^	137.2 ± 46.0	123.5 ± 35.4	182.1 ± 40.3	143.6 ± 35.7
Apo-B (mg/dL)^*∗*^	80.0 ± 31.1	120.5 ± 47.5	127.2 ± 51.1	149.8 ± 89.5
FFA (mmol/mL)	2.5 ± 1.0	3.1 ± 2.2	3.1 ± 2.3	3.1 ± 1.9
CRP (mg/L)^*∗*^	4.47 ± 2.11	6.83 ± 3.52	6.29 ± 3.83	7.4 ± 3.7
C3 (mg/dL)^*∗*^	110 ± 30	137 ± 38	116 ± 25	135 ± 26
ESR (mm/h)	6 ± 4	10 ± 7	12 ± 8	12 ± 5

*n* = 151. Data are presented as mean ± SD. ^*∗*^Mann-Whitney *U* test with *P* < 0.05 comparing the groups by age: individuals with abdominal obesity versus individuals without abdominal obesity. HDLc, LDLc, and VLDLc: high density lipoprotein cholesterol, low density lipoprotein cholesterol, and very low density lipoprotein cholesterol, respectively; Apo: apolipoprotein; CRP: C reactive protein; FFA: free fatty acids; ESR: erythrocyte sedimentation rate.

**Table 3:** Correlation of adiposity with scavenger receptors markers and systemic low-grade inflammatory state along body fat storage.

Measurements	Pearson's correlation test			
	sCD36	*P*	LOX-1	*P*
LOX-1 (pg/mL)	0.249	0.042	—	—
Body mass index (BMI)	**0.359**	**0.003**	−0.359	**0.027**
Waist circumference (cm)	−0.285	**0.008**	−0.276	**0.025**
Hip circumference (cm)	−0.217	**0.045**	−0.188	NS
Total body fat mass (%)	−0.334	**0.020**	−0.221	NS
Trunk body fat mass (kg)	−0.229	**0.036**	−0.225	NS
Total adipose area (cm^2^)	−0.280	**0.009**	−0.262	**0.034**
Visceral area (cm^2^)	−0.282	**0.009**	−0.251	**0.042**
Sagittal diameter (cm)	−0.265	**0.014**	−0.261	**0.033**
Body fat index	−0.301	**0.038**	−0.612	**0.001**
Waist-to-height ratio (WHtR)	−0.313	**0.004**	−0.378	**0.019**
Conicity index	−0.256	**0.019**	−0.371	**0.022**
Skinfold thickness (mm)				
Bicipital	−0.283	**0.008**	−0.255	**0.037**
Subscapular	−0.308	**0.004**	−0.235	NS
Suprailiac	−0.305	**0.004**	−0.251	**0.040**
Abdominal	−0.325	**0.002**	−0.258	**0.036**
ST5 (mm)	−0.257	**0.017**	−0.245	**0.048**
Basal glucose (mg/dL)	−0.189	NS	−0.258	**0.038**
Total cholesterol (mg/dL)	−0.255	**0.017**	0.001	NS
HDLc (mg/dL)	−0.292	**0.044**	0.089	NS
LDLc (mg/dL)	−0.247	**0.021**	−0.027	NS
VLDLc (mg/dL)	−0.379	**0.009**	−0.406	**0.013**
LDLc/HDLc	−0.251	**0.019**	−0.053	NS
Apo-A1 (mg/L)	0.463	**0.001**	−0.383	**0.018**
Apo-A1/Apo-B	−0.225	**0.039**	−0.048	NS
FFA (mmol/mL)	0.377	**0.020**	0.167	NS
CRP (mg/L)	−0.325	**0.002**	−0.072	NS
C3 (mg/dL)	−0.324	**0.024**	−0.431	**0.007**
ESR (mm/h)	−0.107	NS	−0.396	**0.014**

*n* = 151. HDLc, LDLc, and VLDLc: high density lipoprotein cholesterol, low density lipoprotein cholesterol, and very low density lipoprotein cholesterol, respectively; Apo: apolipoprotein; CRP: C reactive protein; FFA: free fatty acids; ESR: erythrocyte sedimentation rate.
